# Para-occupational exposure to pesticides, *PON1* polymorphisms and hypothyroxinemia during the first half of pregnancy in women living in a Mexican floricultural area

**DOI:** 10.1186/s12940-019-0470-x

**Published:** 2019-04-11

**Authors:** Luisa Torres-Sánchez, Ricardo Gamboa, Susana Bassol-Mayagoitia, Claudia Huesca-Gómez, Martha Patricia Nava, Jennifer Illian Vázquez-Potisek, Leticia Yáñez-Estrada, Rebeca Mejía-Saucedo, Julia Blanco-Muñoz

**Affiliations:** 1 Instituto Nacional de Salud Pública, Av. Universidad 655, Col. Sta. María Ahuacatitlán, 62100 Cuernavaca, Morelos, CP Mexico; 2 Physiology Department, Instituto Nacional de Cardiología Ignacio Chávez, Juan Badiano 1, 14080 Mexico City, C.P Mexico; 3 Faculty of Medicine, Universidad Autónoma de Coahuila, Morelos 900, 27000 Torreón, Coahuila, C.P Mexico; 4 Faculty of Medicine, Universidad Autónoma de San Luís Potosí, Venustiano Carranza 2405. San Luis Potosí, 78000 San Luís Potosí, C.P Mexico

**Keywords:** PON1 polymorphisms, Thyroid hormones, Pregnancy, Pesticides

## Abstract

**Background:**

Adequate maternal thyroxine (T4) concentrations during the first half of pregnancy are fundamental to the embryo’s or fetus’ neural development. Organophosphate pesticides (OP) can act as thyroid disruptors and genetic polymorphisms for paraoxonase 1 (PON1), an enzyme that detoxifies OP, could be involved in individual’s susceptibility to them. We assessed the association between para-occupational exposure to pesticides, including OP, during pregnancy and maternal hypothyroxinemia, as well as the potential genetic susceptibility conferred by *PON1* polymorphisms.

**Methods:**

We analyzed information from 381 healthy pregnant women (< 17 gestational weeks), who lived in a floricultural region of Mexico where pesticides, including OP, are routinely used. Women who were para-occupationally exposed to pesticides were those whose partner had an occupation involving contact with these products. Thyroid-Stimulating Hormone (TSH) and free T4 concentrations were determined using ELISA, and hypothyroxinemia was defined as free T4 concentrations <0.76 ng/dL. *PON1192QR*, *PON155LM* and *PON1–108CT* polymorphisms were determined through Polymerase Chain Reaction (PCR). The association between para-occupational exposure and genetic polymorphisms and hypothyroxinemia was estimated using logistic regression models.

**Results:**

One hundred and sixty two women (42.52%) were classified as para-occupationally exposed to pesticides. Hypothyroxinemia prevalence was 54%, and it was not significantly associated with pesticide para-occupational exposure (OR: 1.21 95% CI 0.75–1.94). Independently of para-occupational exposure, the likelihood of hypothyroxinemia was higher among women who were carriers of *PON155MM* than in those with *PON155LL* genotype (OR _MM vs LL_: 3.03; 95%CI 1.62, 5.70). *PON1192 RR* (OR _RR vs QQ_: 1.72; 95%CI 0.93, 3.17) and *PON1–108TT* (OR _TT vs CC_: 1.60; 95%CI 0.90, 2.70) genotypes were marginally associated with hypothyroxinemia. No significant interaction was observed between pesticides para-occupational exposure and *PON1* polymorphisms.

**Conclusions:**

These results suggest that *PON1* polymorphisms could affect thyroid function during pregnancy in women living in areas where pesticides, including OP, are routinely used. Low exposure variability in this population, could be a possible explanation for the lack of association between para-occupational exposure and thyroid function.

## Introduction

Thyroid hormones play a fundamental role in cellular metabolism and in nervous system growth and development, especially during the intrauterine period and early childhood. The fetal thyroid gland efficiently produces thyroid hormones at approximately 16–18 weeks of pregnancy, meaning that during the first half of pregnancy the embryo or fetus requires an adequate maternal thyroid function for adequate neuronal migration and differentiation [1]. Thyroxine (T4) can cross the placental barrier and moderate and severe maternal hypothyroxinemia during the first weeks of pregnancy has been associated with infant neurodevelopment alterations [2, 3].

Iodine deficiency and autoimmune disease are the factors most frequently involved in thyroid dysfunction; however, the effect of environmental toxins exposure on thyroid function has become relevant in the last decades [4]. Evidences from the general population [5] and from men of reproductive age [6, 7], suggest that exposure to organophosphate pesticides can produce subclinical changes of the thyroid profile. Nevertheless, its importance at a clinical or biological level is not clearly established and few studies have included factors related to genetic susceptibility to these toxins [8].

Paraoxonase 1 (PON1) is an enzyme linked to high density proteins that decreases lipid peroxidation [9] and is also involved in detoxification of some OP pesticides. The gene that codes for PON1 has two common polymorphisms in the coding region. The first is a substitution of methionine for leucine in position 55 (*55 L/M*), and the second one is a substitution of arginine for glutamine in position 192 (*192Q/R*). There are other polymorphisms in the promoter region, of which the most studied is *-108C/T*. The *T* allele at position − 108 and the *M* allele in position 55 are associated with plasmatic paraoxonase level decrease [10]; while the *R* allele in position 192 synthesizes a less efficient paraoxonase enzyme against HDL and LDL oxidation [11]. PON1’s detoxifying activity depends on enzyme structure and substrate. Both isoforms resulting from *PON1*_*192*_ polymorphism have a different hydrolysis rate against some OP substrates: *PON1*_*192R*_ metabolizes chlorpyrifos pesticide more efficiently than isoform *PON1*_*192Q*_, whereas no difference was observed in diazinon metabolism [12].

A study performed on healthy post-menopausal women with no pesticide exposure found a negative association between *R* allele of the polymorphism *PON1*_*192*_ and free T3 levels [13]. Additionally, in male flower workers from a floricultural area of Mexico, our research group found a positive association between urine concentration of dialkylphosphate (DAP) OP metabolites and TSH and total T4 levels and a negative association with total T3 [6], as well as a decrease in the percentage of variation of TSH among carriers of *PON1*_*192*_*RR* genotype [8]. Among pregnant women residing in this area we found a high frequency of hypothyroxinemia (53%) [14].

Given the importance of maternal thyroid function in adequate fetal development and the scarcity available information on pregnant women, in the present study we estimated the association between para-occupational exposure (*exposure of a worker’s family to substances carried from the workplace to the home*) to pesticides and maternal hypothyroxinemia during the first half of pregnancy, as well as the possible interaction with maternal *PON1* polymorphisms (*PON1192Q/R, PON155 L/M and PON1–108C/T*).

## Material and methods

### Study population and data collection instruments

We assembled a pregnancy cohort study in a floricultural region of the State of Mexico, the main flower producing area in the country, located in central Mexico. The main objective was to evaluate the effect of pesticide exposure during pregnancy, as well as the interaction with PON1 polymorphisms, on the occurrence of adverse reproductive outcomes, with a particular interest in maternal thyroid profile. Details about the selection of the study population have been published previously [14].

Briefly, during the period of July 2013–August 2015, we identified pregnant women who were 15–40 years old, with a gestational age of less than 17 weeks, with no chronic illnesses, and a residence time of at least 1 year in Tenancingo, Zumpahuacán, Tonatico and Villa Guerrero. In these municipalities, the main economic activity is floriculture, and the inhabitants are environmentally, para-occupationally or occupationally exposed to pesticides, mainly those of the OP family: diazinon, methamidophos, omethoate and methyl parathion [15].

Eligible women were identified at health centers of these municipalities when they attended for their first prenatal visit. All participating women signed an informed consent letter; for women under 18 years of age, in addition to their agreement we obtained an informed consent letter from their parents. The study was approved by the Ethics Committee of the National Public Health Institute of Mexico.

Once women were recruited, height and weight were measured and they were asked to complete a questionnaire to collect sociodemographic characteristics, reproductive background, work history, alcohol, tobacco and coffee consumption before and during pregnancy and intake of supplements containing iodine during 3 months prior to becoming pregnant and until the time when they were included in the study. They were also queried regarding the child’s father (sociodemographic data, medical history, and occupation). To determine thyroid hormones (TH), *PON1192*, *PON155*, and *PON1–108* genotypes, and OP metabolites, a blood and urine sample was obtained under fasting conditions between 8 and 10:30 AM from each woman.

Out of 635 eligible pregnant women, 480 (75.6%) agreed to participate in the study. The main reason for non-participation was lack of time. This report corresponds to 381 pregnant women who had complete questionnaire information, and whose *PON1* polymorphisms and TH results at the time they entered the study (baseline) were available. No significant differences were observed between these women and the remaining participants with respect to sociodemographic characteristics, number of pregnancies, alcohol and tobacco use, and gestational age at baseline (data not shown).

### Thyroid hormones determination

Serum TSH concentrations and free T4 were determined by Enzyme-Linked ImmunoSorbent Assay (ELISA), using an automated immunoassay system (DRG International, Inc., USA). Reference values for TH concentrations for pregnant women were assessed according to the information provided in the commercial kits, as follows: a) TSH 0.5 to 5.0 mlU/L and free T4 0.76 to 2.24 ng/dL.

Intra-assay serum TSH variation coefficients and free T4 were 3.36 to 3.88% and 3.2 to 10.9%, respectively. Inter-assay variation coefficients were 3.3 to 9.1% and 7.9 to 10.8%, respectively. Analytical sensitivity for TSH and free T4 were 0.06 mIU/L and 0.05 ng/dL, respectively.

Since TSH levels decrease physiologically during pregnancy, we categorized them following the American Thyroid Association (ATA) criteria [16], which consider a normal range of 0.1–4 mIU/L for pregnant women, and concentrations > 4 mlU/L to be suggestive of hypothyroidism.

Hypothyroxinemia was defined as a free T4 lower than 0.76 ng/dL. So, isolated hypothyroxinemia was defined as TSH concentrations ≤4 mlU/L and a free T4 concentration < 0.76 ng/dL. Subclinical hypothyroidism was defined as TSH concentrations > 4mlU/L, and a free T4 concentration ≥ 0.76 ng/dL. Overt hypothyroidism was considered if TSH concentrations > 4 mlU/L and free T4 concentration < 0.76 ng/dL. Only three participants had subclinical hypothyroidism and seven had overt hypothyroidism. Therefore, we decided to create a variable with two categories: “Women with hypothyroxinemia”—which included women with isolated hypothyroxinemia, women with subclinical hypothyroidism and women with overt hypothyroidism— and “Women without hypothyroxinemia”.

### Pesticide exposure

Due to financial reasons, we did not measure OP exposure biomarkers in all women; instead, we decided to use the partner’s occupation as a proxy for pesticide exposure, including OP. A recent review of non-occupational pathways for pesticide exposure in women living in agricultural areas found that farm homes (where one or more family members use or contact pesticides at work) had higher concentrations of pesticides in residential dust than non-farm homes, whereas the results from biomonitoring studies were often difficult to interpret because of low detection rates or limited variability in pesticide biomarkers [17]. Previously, we found that workers frequently take the pesticide spraying equipment home, store pesticides and bring work shoes and clothes into the house, providing an important source of para-occupational exposure for their families [18]. Those women who reported that their husband worked in agricultural activities or transported or sold pesticides were considered para-occupationally exposed to them, while those whose partners’ occupations did not involve exposure to these products were considered “not para-occupationally exposed”. We implicitly assumed that para-occupationally exposed women would be more exposed to pesticides than not para-occupationally exposed women. Only two women were occupationally exposed to pesticides, as were their partners, so they were classified as “para-occupationally exposed”.

To verify the previous assumption, we selected a random subsample of 65 women (20 of them were classified as para-occupationally and 45 as no para-occupationally exposed to OP by questionnaire), and we measured the urinary six common dialkylphosphate (DAP) metabolites of organophosphate pesticides: dimethylphosphate (DMP), dimethylthiophosphate (DMTP), dimethyldiithiophosphate (DMDTP), diethylphosphate (DEP), diethylthiophosphate (DETP), and diethyldithiophosphate (DEDTP). Metabolites were determined by gas–liquid chromatography with Electron Capture Detector (GC-ECD). These metabolites are non-specific to a particular organophosphate but they are useful to estimate exposure to several organophosphates [19]. The dimethyl (DMP, DMTP, and DMDTP) and diethyl (DEP, DETP, and DEDTP) metabolite concentrations were converted to their molar concentrations (μmol/l) and summed to produce total DAP concentration for each sample. Metabolite concentrations were adjusted using creatinine concentration to correct for variable urine dilutions. Urinary creatinine concentration was determined by spectrophotometry using a commercial kit (Randox Creatinine Kit).

### Determining PON1 192QR, 55LM and -108CT polymorphisms

Genomic DNA was isolated from peripheral blood using commercial kits + (Promega). DNA was quantified using a spectrophotometer at a 260/280 nm wavelength. Extraction purity was measured. Genotypes were determined using the TaqMan® PCR assay (Applied Biosystems). When the specific fluorogenic probes of each allele are hybridized to the template during PCR, Taq polymerase is able to discriminate alleles. Excision results in a higher emission of a coloring agent. Each assay requires two unmarked PCR primers and two allele-specific probes. Each probe is marked with a coloring agent for identification (VIC and FAM). TaqMan real time PCR was used to detect polymorphism sites -108C/T (rs: 705379), 192Q/R (RS: 662), and 55 L/M (rs: 854560) in an automatic sequencing machine (ABI Prism 7000) according to the manufacturer’s directions (Applied Biosystems, Foster City, CA, USA.). Discrimination was used for each studied genotype and allele, both manually and automatically, using an allelic discrimination software (7300 Sistema SDS Software® by Applied Biosystems).

To evaluate the precision and fidelity of the test, we reviewed the results at two different time point’s moments by the same molecular biologist and also evaluated the results through intra-observer and between inter-observer.

### Description of covariates

As potential confounders, variables considered relevant due to biological reasons or reported to be associated with the outcome were evaluated [20]: maternal age (years); gestational age (weeks) and body mass index (BMI) [kg/m^2^] at the time of the first interview, all of them were analyzed as continuous variables in regression models; other evaluated variables were: maternal education (categorized as elementary school, middle school and more than middle school), consumption of one or more cups of coffee per day during the pregnancy (Yes or No), active smoking and alcohol intake at some time during the individual’s life, including the current pregnancy (Yes or No), consumption of iodine supplements from the 3 months before pregnancy to the date of the interview (Yes or No). Because a circadian and circannual variation in TH concentration has been reported [21, 22], we also included the time of the day (in hours) and season of the sample-taking. We operationalized the “season” variable in a dichotomous manner, according to changes in Mexican summer time (April to September) and winter time (October to March).

### Statistical analysis

TSH and free T4 concentrations were described using means and percentiles. Both TH were not normally distributed, and thus the geometric means were calculated. Allelic and genotype frequencies were calculated by direct counting. The χ^2^ test was used to determine genotype frequency deviation from Hardy–Weinberg (H–W) equilibrium expectations and to evaluate the significance of the linkage disequilibrium between the polymorphisms.

Women with or without hypothyroxinemia were compared by selected characteristics. In the case of categorical variables we used the χ^2^ test, while Student’s t test or non-parametric tests (Mann-Whitney test) were used for continuous variables.

Total DAP concentrations available for 65 randomly selected women, were compared according to the para-occupational categories, by means of the non-parametric Mann-Whitney test.

The association of para-occupational exposure and each studied polymorphism, with hypothyroxinemia, was assessed by simple and multiple logistic regression models. *PON1* genotypes were examined categorically and the wild genotype (*PON1192QQ, PON155LL, PON1–108CC*) of each one was considered as the reference. All final models were adjusted by maternal age, gestational age and BMI. We also kept those variables that modified the association between the independent variables of interest and hypothyroxinemia by more than 10% (maternal education and coffee intake).

To assess potential interactions between para-occupational pesticide exposure and *PON1* polymorphisms, interaction terms were included in multivariate models. Interaction was considered significant if *p* value was < 0.15.

Two sensitivity analyses were performed, one excluding the ten women with overt or subclinical hypothyroidism and another one excluding the two women occupationally exposed. We also carried out a sub- analysis evaluating the association between total DAP exposure and hypothyroxinemia in the subsample of 65 women with urine biomarkers.

Statistical analysis was performed with Stata 14.0 statistical software.

## Results

In the whole group, 162 women (42.52%) were classified as para-occupationally exposed to pesticides. In the subsample of women with available DAP information (*n* = 65), no significant difference was observed in the median of total urinary DAP concentration between those para-occupationally exposed (*n* = 20) and those no para-occupationally exposed (*n* = 45): 1.16 vs. 0.83 μmol/gr creatinine, *p* = 0.56. (Data not shown in Tables).

A total of 207 women (54%) had free T4 concentrations lower than 0.76 ng/dL and 10 women had TSH > 4 mUI/L. Out of these women, 197 had isolated hypothyroxinemia, three had subclinical hypothyroidism and seven had overt hypothyroidism (Table 1). As expected, TSH concentrations in women with hypothyroxinemia were significantly higher than in women without hypothyroxinemia (1.40 ± 1.50 vs. 1.04 ± 0.97 mIU/L, *p* < 0.01) (Data not shown in tables).

**Table 1:** Maternal thyroid hormone levels during the first 16 weeks of pregnancy

Hormone	*n* = 381	(%)	Mean ± SD^a^	P_10_^c^	P_50_^c^	P_90_^c^
Free T4(ng/dL) ^b^			0.79 ± 0.34	0.48	0.75	0.96
< 0.76	207	54.3				
≥ 0.76	174	45.7				
TSH (mUI/L) ^b^			1.24 ± 1.30	0.22	0.87	2.58
≤ 4	371	97.4				
> 4	10	2.6				
Overt hypothyroidism^d^	7	1.8				
Sub-Clinical hypothyroidism^d^	3	0.8				
Isolated Hypothyroxinemia^d^	197	51.71				
Without Hypothyroxinemia^e^	174	45.67				

^a^Arithmetic mean and Standard Deviation (SD)

^b^Geometric mean and SD: TSH = 0.83 ± 0.8 mUI/L; Free T4 = 0.75 ± 0.3 ng/dL

^c^10, 50 and 90 percentiles

^d^Hypothyroxinemia includes: Overt hypothyroidism (TSH concentrations > 4 mlU/L and a free T4 concentration < 0.76 ng/dL); Subclinical hypothyroidism (TSH concentrations >4mlU/L, and a free T4 concentration ≥ 0.76 ng/dL); Isolated hypothyroxinemia (TSH concentrations ≤4 mlU/L and a free T4 concentration < 0.76 ng/dL)

^e^Without hypothyroxinemia: a free T4 concentration ≥ 0.76 ng/dL)

Women with hypothyroxinemia were older (24.83 ± 5.98 vs. 23.45 ± 5.26; *p* = 0.02), had a higher frequency of elementary school education (25.6% vs. 13.2%; *p* = 0.001) and less history of tobacco use (27.5% vs. 37.93%; *p* = 0.03) than women without hypothyroxinemia. A higher frequency of blood samples obtained from women that were classified as hypothyroxinemic were taken in summer (52.17% vs 41.95%; *p* = 0.05). No other significant differences were observed (Table 2).

**Table 2:** Selected characteristics of pregnant women according to hypothyroxinemia condition

Characteristics	All women	Hypothyroxinemia		*p* value^a^
	*N* = 381	Yes *n* = 207	No *n* = 174	
Maternal Age (years)				
Mean ± SD	24.20 ± 5.7	24.83 ± 5.98	23.45 ± 5.26	0.02
Marital status n(%)				
United^b^	338 (88.71)	188 (90.82)	150 (86.21)	0.16
No united	43 (11.29)	19 (9.18)	24 (13.79)	
Education n(%)				
Elementary school	76 (19.95)	53 (25.60)	23 (13.22)	< 0.01
Middle school	189 (49.61)	105 (50.72)	84 (48.28)	
> Middle school	116 (30.45)	49 (23.67)	67 (38.51)	
Gestational age (weeks)				
Mean ± SD	10.20 (2.80)	10.36 ± 2.83	9.99 ± 2.74	0.20
Body Mass Index^c^				
Mean ± SD	24.50 (4.40)	24.85 ± 4.46	24.07 ± 4.30	0.08
Coffee intake n(%)				
Yes	293 (76.90)	153 (73.91)	140 (80.86)	0.13
No	88 (23.10)	54 (26.09)	34 (19.54	
Parity n(%)				
Primiparous	157 (41.21)	79 (38.16)	78 (44.83)	0.19
Multiparous	224 (58.79)	128 (61.84)	96 (55.17)	
Smoking n(%)				
Yes	123 (32.28)	57 (27.54)	66 (37.93)	0.03
No	258 (67.72)	150 (72.46)	108 (62.07)	
Alcohol intake n(%)				
Yes	253 (66.40)	137 (66.18)	116 (66.67)	0.92
No	128 (33.60)	70 (33.82)	58 (33.33)	
Iodine supplementation^d^ n(%)				
Yes	43 (11.91)	24 (12.18)	19 (11.59)	0.86
No	318 (88.09)	173 (87.82)	145 (88.41)	
Sampling season n(%)				
Summer	181 (47.51)	108 (52.17)	73 (41.95)	0.05
Winter	200 (52.49)	99 (47.83)	101 (58.05)	

^a^χ^2^ or Student t-test, except for BMI, where Mann Whitney test was used

^b^United includes married and women in free union

^c^At time of interview

^d^From 3 months before pregnancy until time of interview

None of the three studied polymorphisms was found to be in Hardy Weinberg equilibrium; no linkage disequilibrium was observed among the three polymorphisms. Genotype and allele distribution of polymorphisms by hypothyroxinemia status was significantly different in the case of polymorphism *PON155LM.* Allele M (42.41 vs. 26.69%; *p* < 0.01) and MM genotype (29.95 vs. 12.07; *p* < 0.01) were more frequent in pregnant women with hypothyroxinemia than in those without hypothyroxinemia. After adjusting for maternal age, gestational age, maternal education, BMI and coffee intake during pregnancy, pesticide para-occupational exposure was not significantly associated with hypothyroxinemia (OR: 1.21 95% CI 0.75–1.94). Hypothyroxinemia during the first half of pregnancy was three times more frequent among carriers of *PON155MM* genotype (OR _*MM* vs *LL*_: 3.03, 95% CI 1.62, 5.7), whereas women with genotypes *PON1192RR* (OR _*RR* vs *QQ*_*:* 1.72, 95%CI 0.93, 3.17) and *PON1–108TT* (OR _*TT* vs *CC*_: 1.60, 95% CI 0.90, 2.74) had a marginally higher likelihood of hypothyroxinemia than women with their respective wild genotypes (Table 3). Independently of para-occupational exposure and maternal genotypes, we also observed a significant decrease in the odds of hypothyroxinemia among those women who reported a higher educational level (OR _≥high school vs elementary school_: 0.28; 95% CI 0.14–0.59; OR _middle school vs elementary school_: 0.51; 95% CI 0.28–0.97; *p* for trend = 0.001) (Data not shown in tables).

**Table 3:** Association of para-occupational exposure to pesticides during pregnancy and PON1 genotypes with maternal hypothyroxinemia

Characteristics	All women *n* = 381(%)	Hypothyroxinemia				p for trend
		Yes *n* = 207 (%)	No *n* = 174 (%)	Crude OR (95%CI)	Adjusted OR (95%CI)	
Para-occupational exposure to pesticides during pregnancy^a^						
No	219 (57.48)	113 (54.59)	106 (60.92)	1.0	1.0	
Yes	162 (42.52)	94 (45.41)	68 (39.08)	1.35 (0.88–2.06)	1.21 (0.75–1.94)	
*PON1 192QR* ^*b*^						
QQ	119 (31.23)	62 (29.95)	57 (32.76)	1.0	1.0	0.11
QR	161 (42.26)	82 (39.61)	79 (45.40)	0.89 (0.54–1.45)	0.87 (0.52–1.47)	
RR	101 (26.51)	63 (30.43)	38 (21.84)	1.68 (0.95–2.96)	1.66 (0.92–2.99)	
Q allele	399 (52.30)	206 (49.75)	193 (55.39)	1		
R allele	363 (47.55)	208 (50.12)	155 (44.48)	1.31 (0.98–1.77)	1.30 (0.96–1.77)	
PON155LM*^b^						
LL	195 (51.18)	93 (44.93)	102 (58.62)	1.0	1	0.001
LM	103 (27.03)	52 (25.12)	51 (29.31)	0.95 (0.58–1.55)	1.10 (0.65–1.84)	
MM	83 (21.78)	62 (29.95)	21 (12.07)	2.47 (1.37–4.45)	3.01 (1.63–5.57)	
L allele	493 (64.58)	238 (57.35)	255 (73.18)	1	1	
M allele	269 (35.23)	176 (42.41)	93 (26.69)	1.67 (1.21–2.29)	1.93 (1.38–2.69)	
PON1–108CT*^b^						
CC	130 (34.12)	61 (29.47)	69 (39.66)	1.0	1	0.21
CT	140 (36.75)	83 (40.10)	57 (32.76)	1.36 (0.82–2.24)	1.36 (0.81–2.31)	
TT	111 (29.13)	63 (30.43)	48 (27.59)	1.38 (0.81–2.35)	1.42 (0.82–2.44)	
C allele	400 (52.48)	205 (49.40)	195 (55.96)	1	1	
T allele	362 (47.42)	209 (50.36)	153 (43.91)	1.23 (0.92–1.66)	1.25 (0.92–1.70)	

^a^Model adjusted by: maternal age, education, gestational age, coffee intake, body mass index and maternal PON 1 Genotypes

^b^Model adjusted by: maternal age, education, gestational age, coffee intake, body mass index and para-occupational exposure to pesticides during pregnancy

*D’ between PON155LM and PON1–108CT polymorphisms *p* = 0.82; Hardy–Weinberg equilibrium for all polymorphisms: *p* < 0.05

Although we did not observe significant interactions between pesticide para-occupational exposure during pregnancy and the genetic polymorphisms, among women para-occupationally exposed, carriers of *PON1–108TT* genotype, the odds of hypothyroxinemia were more than two times greater than those observed among carriers of *-108CC* genotype (Table 4). Similar results were observed when we included all polymorphisms in the model (Data not shown in tables).

**Table 4:** Models results for the interaction between para-occupational pesticide exposure during pregnancy and PON1 genotypes on maternal hypoythyroxinemia

Maternal PON 1 Genotype		Para-occupational exposure during pregnancy				
		Yes		No		
	n	Crude OR (95% CI)	Adjusted OR^a^ (95% CI)	n	Crude OR (95% CI)	Adjusted OR^a^ (95% CI)
*PON1192*						
QQ	55	1.10 (0.52–2.34)	0.97 (0.44–2.15)	64	1.0	1.0
QR	66	1.27 (0.47–3.43)	1.28 (0.45–3.62)	95	0.81 (0.42–1.56)	0.78 (0.75–1.94)
RR	41	1.61 (0.50–5.14)	1.52 (0.46–5.04)	60	1.41 (0.67–2.96)	1.39 (0.64–3.0)
*p for interaction:* 0.78						
*PON155*						
LL	82	1.13 (0.63–2.04)	1.07 (0.58–1.99)	113	1.0	0.94–1.091.0
LM	45	1.31 (0.48–3.56)	1.15 (0.41–3.25)	58	0.84 (0.44–1.62)	1.02 (0.51–2.05)
MM	35	1.87 (0.55–6.38)	1.49 (0.42–5.27)	48	1.96 (0.94–4.09)	2.59 (1.21–5.57)
*p for interaction:* 0.82						
*PON1108*						
CC	54	0.85 (0.41–1.76)	0.70 (0.32–1.49)	76	1	1.0
CT	62	1.98 (0.72–5.48)	2.11 (0.73–6.10)	78	1.00 (0.52–1.95)	0.98 (0.48–1.49)
TT	46	2.11 (0.71–6.33)	2.49 (0.79–7.81)	65	1.02 (0.51–2.04)	0.96 (0.47–1.98)
*p for interaction:* 0.23						

^a^Models were adjusted by: maternal age, gestational age, maternal education, coffee intake, and body mass index

These results did not change when we excluded the 10 women with overt and subclinical hypothyroidism or the two occupationally exposed women (data not shown). Likewise, regarding hypothyroxinemia and pesticides exposure, no association (OR = 1.00, 95% CI 0.99–1.004) was observed in the subsampled women when we used as exposure biomarker, the urinary total DAP concentrations. (Data not shown in tables).

## Discussion

The results of this study do not point to an association between para-occupational exposure to pesticides and maternal hypothyroxinemia in the first half of pregnancy. However, *PON155MM* genotype increased the frequency of hypothyroxinemia, independently of para-occupational exposure. Para-occupational exposure seems to be related to hypothyroxinemia, among women carriers of *PON1–108 T* allele, but the interaction between genotype and exposure was not statistically significant.

Biological mechanisms that might explain the independent association between PON1 polymorphisms and hypothyroxinemia are not clear, but a possible hypothesis is through the antioxidant ability of PON1 [13]. Both *PON1* polymorphisms and thyroid function have been independently related to oxidative stress [23, 24]. PON1 is an antioxidant enzyme that prevents the peroxidation of LDL and in myocardial infarct patients, a negative association was observed between PON1 concentrations and malonyl aldehyde (MDA) concentrations, an oxidative stress marker [23]. Moreover, polymorphism *192Q/R* has been associated with a higher frequency of coronary spasm and higher plasmatic concentrations of another oxidative stress marker, thiobarbituric acid-reactive substances (TBARS) [25]. In psoriasis patients, whose pathogenesis has also been linked to oxidative stress, it was also found that *PON155M* allele carriers have significantly higher MDA concentrations than *PON155L* allele carriers [26].

An adequate equilibrium between oxidation products and antioxidant enzymes is necessary for correct thyroid function. Alterations in this balance can be due to an increase in oxidant production or a decreased quality or concentration of antioxidant enzymes [13]. Physiologically, pregnancy increases oxygen demand and, consequently, Reactive Oxygen Species (ROS) production, as well as lipid peroxidation [27]. ROS and free radicals can have both beneficial and detrimental effects on the thyroid gland. Thus, although hydrogen peroxide is essential to thyroid hormone production, as it is a co-factor for iodization of T4 precursors, ROS increase can cause thyroid dysfunction [28, 29]. In patients with kidney insufficiency who were not undergoing dialysis, it was found that MDA increase was associated with a T3 and T4 decrease [30]. Similarly, fever has been associated with increased production of oxidative stress markers, such as TBARS and MDA, and with decreased 5-monoiodinase activity, which in turn decreases T3-to-T4 conversion [31]. It has also been observed that oxidative stress inhibits transcription factors bonding to thyroid differentiation genes [32]. Since pregnancy is a pro-oxidant state, reduced PON1 activity or concentration associated with some *PON1* polymorphisms could alter this equilibrium between oxidation products and antioxidant enzymes, affecting thyroid function.

Genotype and allele distributions of polymorphisms *PON1192QR* and *PON155LM* are consistent with the results found in a racially mixed population in Mexico, where alleles *PON1192Q* and *PON155L* were the most frequent (52.2 and 84.6%, respectively) [33]. In our study population none of the three studied polymorphisms was found to be in Hardy Weinberg equilibrium; this could be due to the fact that the inhabitants are in closed villages, with a small number of inhabitants and in which it is common to find that marriages occur among the inhabitants of the same community, which would limit genetic diversity. TSH concentration was significantly higher in women with hypothyroxinemia, compared to women without hypothyroxinemia, which is consistent with the physiological feedback response of the hypothalamus-hypophysis-thyroid axis.

It is difficult to compare our results to those of other researchers, because few epidemiological studies have explored the effects of pesticide exposure on the thyroid profile of pregnant women. Most of them have evaluated the effect of persistent organochlorine compounds, founding inconsistent results [14]. Zhang et al., in Japan, found no association between urinary pyrethroid concentrations and maternal thyroid profile during the first trimester of pregnancy [34], nor with the infant’s thyroid profile at birth [35]; however, these results have been questioned due to possible selection bias [4]. A cohort study performed in Denmark on women who worked in greenhouses during pregnancy and were classified as moderately/highly exposed or not exposed to pesticides (in general), found a negative association between prenatal exposure to these compounds and the TSH concentrations of their children 6–11 years of age (β = − 0.56, 95%CI -1.287, − 0.022), but the effect upon maternal thyroid function during pregnancy was not evaluated [36].

In a previous study performed on male flower workers from the geographical area of the present study, we found a significant interaction between DAP concentrations and polymorphism *PON1192QR*, upon serum TSH levels, and to a lesser extent, upon T3 levels. No interaction was observed with polymorphism *PON155LM* [8]. Nonetheless, comparability between studies is limited, given that our population includes pregnant women who are mainly para-occupationally exposed to pesticides and exposure biomarkers were not available. We only found a single previous study, performed on healthy post-menopausal women not exposed to pesticides, that assessed the effect of *PON1192QR* polymorphism on the thyroid profile finding that *R* allele carriers had significantly lower free T3 concentrations than women with *QQ* genotype (homozygous *QQ*: 2.46 pg/ml, heterozygous 2.05 pg/ml, homozygous *RR*: 2.16 pg/ml; *p* = 0.047) [13].

For the correct interpretation of our results, we must take into account some limitations: We cannot reject a potential misclassification error because we assumed that pregnant women, whose partners worked in a job involving pesticide use, would be more exposed to OP and other pesticides than women with partners who held a different type of job. However, all women resided in localities where floriculture is one of the main economic activities and OP are one of the most used pesticides; thus, it is possible that exposure levels of para-occupationally exposed women are quite similar to those of the rest of the study population. Nonetheless the possibility that our results could be a consequence of a differential measurement error is low, because exposure data were obtained from all women before TH determination, and whoever performed the thyroid hormone determination was unaware of the women’s exposure status. A possible non-differential measurement error along with a low variability in exposure levels among our women could explain the lack of significant association between para-occupational exposure and hypothyroxinemia.

Additionally, we had no information on PON1 concentration and enzyme activity. This is relevant because even among individuals with the same genotype, PON1 activity can vary up to 13 times [37].

On the other hand, differences in the time and season of sampling as well as iodine bioavailability could affect TH concentrations. For these reasons, we adjusted by the time and season of sample-collection and by iodine supplementation but this did not confound the results of association between para-occupational exposure to pesticides or *PON1* polymorphisms and hypothyroxinemia.

We found a high frequency of isolated hypothyroxinemia in our study population. According to a recent review, which included studies that measured the free T4 concentration by immunoassay, the prevalence of isolated hypothyroxinemia varied between 1.3 and 24% during pregnancy; the authors attribute this wide variation to the ethnic differences and iodine bioavailability of the studied populations, as well as to cut-off points selection [38]. The American Thyroid Association [16] has proposed that reference ranges for free T4 during pregnancy should be established for each specific population and trimester of pregnancy, in samples of women without thyroid disease, without thyroid antibodies and with adequate iodine consumption. Following these criteria, in Mexico, we only have found a study conducted in Yucatan [39] which established a reference range for the first trimester from 0.75 to 1.7 ng/dL, not far from the range proposed by our laboratory (0.76 to 2.24 ng/dL). A recent study including Mexican pregnant women without history of thyroid disease, reported that the prevalence of isolated hypothyroxinemia was 12.8%; however, the whole frequency of thyroid disorders was 46.7% [40]; this suggests that prevalence of thyroid disorders during pregnancy in Mexico, could be greater than that reported by literature. Given that the prevalence of iodine deficiency is low in our study population [14], immunological factors and exposure to other endocrine disruptors as synthetic fertilizers that include nitrites in their composition, could explain the high frequency of isolated hypothyroxinemia we find, a hypothesis that should be evaluated in future studies.

## Conclusion

In these women living in areas where pesticides are routinely used, our findings suggest that *PON1 55MM* genotype is independently associated with maternal hypothyroxinemia during the first half of pregnancy. Since there is scarce literature on the subject, additional epidemiologic studies are required to confirm or refute our results. Basic research oriented towards elucidating the biological mechanisms through which *PON1* polymorphisms could be involved in thyroid disruption in pro-oxidant conditions like pregnancy is also needed.

We found no association between hypothyroxinemia and para-occupational exposure, however we cannot claim that there is no correlation between hypothyroxinemia and OP pesticide exposure, because it is quite possible that the control group in this study is also highly exposed through other pathways.

## Abbreviations

BMI: Body Mass Index
DAP: Dialkylphosphate metabolites
OP: Organophosphate pesticides
PCR: Polymerase Chain Reaction
PON1: Paroxonase 1
T4: Thyroxine
TSH: Thyroid-Stimulating Hormone
